# A randomized, blinded, controlled, multi-centered field study assessing the treatment of gastrointestinal nematode infections in cats with fluralaner plus moxidectin spot-on solution (Bravecto® Plus)

**DOI:** 10.1186/s13071-018-3169-x

**Published:** 2018-11-19

**Authors:** Nadja Rohdich, Eva Zschiesche, Oliver Wolf, Wolfgang Loehlein, Zvezdelina Kirkova, Petar Iliev, Dhimitër Rapti, Rezart Postoli, Balázs Capári, Róbert Farkas, Rainer K. A. Roepke

**Affiliations:** 1 MSD Animal Health Innovation GmbH, Zur Propstei, 55270 Schwabenheim, Germany; 2 Loehlein & Wolf Vet Research, Maistrasse 69, 80337 Munich, Germany; 3 Department of Parasitology, Trakia University, Faculty of Veterinary Medicine, Student Campus, 6000 Stara Zagora, Bulgaria; 4 Klinika Veterinare Xhimi, Bulevardi Bajram Curri Nr 2, Tirana, Albania; 5 Faculty of Veterinary Medicine, Agricultural University, Kodër Kamëz, Tirana, Albania; 6 Kapriol Bt, Vak Bottyán St.1, 8330 Suemeg, Hungary; 7 Department of Parasitology and Zoology, Faculty of Veterinary Science, Szent István University, István u. 2, 1078 Budapest VII, Hungary

**Keywords:** Bravecto Plus, Cat, Emodepside, Field study, Fluralaner, Hookworms, Lungworms, Moxidectin, Praziquantel, Roundworms

## Abstract

**Background:**

A spot-on formulation containing fluralaner (280 mg/ml) plus moxidectin (14 mg/ml) (Bravecto® Plus) was developed for the treatment of nematode infections as well as providing 12 weeks of protection against insect and acarine parasites in cats. The effectiveness and safety of this product against feline gastrointestinal nematodes was assessed in naturally-infested, client-owned cats under field conditions in Albania, Bulgaria, Germany and Hungary.

**Methods:**

To be eligible for enrollment in this investigator-blinded study cats had to be at least 10 weeks-old, weigh at least 1.2 kg, be clinically healthy, and have a faecal sample testing positive for nematodes no more than eight days prior to treatment. Cats were stratified into blocks of three in order of presentation at each center and randomly allocated in a 2:1 ratio to be treated topically on Day 0 with fluralaner plus moxidectin (minimum dose rates 40 mg/kg and 2 mg/kg, respectively) or emodepside plus praziquantel (minimum dose rates 3 mg/kg and 12 mg/kg, respectively) (Profender®). Faecal samples were collected from cats prior to treatment and 14 ± 4 days later.

**Results:**

There were 182 cats randomized to the fluralaner plus moxidectin group, and 91 to the emodepside plus praziquantel group. Prior to treatment the most commonly identified nematode egg was *Toxocara cati*, found in 79.1 and 82.4% of cats in the fluralaner plus moxidectin and emodepside plus praziquantel groups, respectively. Eggs of *Toxascaris leonina* were found in 8.2 and 6.6% of cats; of hookworms in 30.8 and 24.2%; and of *Capillaria* spp. in 7.1 and 4.3%, respectively. After treatment, faecal samples from 98.3% of fluralaner plus moxidectin treated and 96.6% of emodepside plus praziquantel-treated cats were free of nematode ova. Geometric mean faecal egg count reductions for *T. cati*, the only eggs found in post-treatment faecal samples, were 99.97% and 99.93%, respectively. Treatment with fluralaner plus moxidectin was non-inferior to emodepside plus praziquantel. Both products were safe and well tolerated by cats treated under field conditions.

**Conclusions:**

This field study confirms that, in addition to 12-week extended duration flea and tick control, fluralaner plus moxidectin provides broad spectrum treatment of nematodes in cats.

## Background

Gastrointestinal nematode infections are common in cats in Europe, with an estimated prevalence of up to 40% [1–7]. Cats are universally at risk of infection, although age and access to the outdoors are clear risk factors. In addition to posing a threat to the health of infected cats, the most commonly occurring nematode, *Toxocara cati*, has zoonotic potential. Therefore, there is a need for vigilance and compliance in the implementation of programs to treat feline nematodes. The European Scientific Counsel Companion Animal Parasites (ESCCAP) recommends that adult cats with access to the outdoors be treated for nematodes at least four times per year [8]. Where there is a higher risk of nematode infection (kittens, adult cats with a predominantly outdoor lifestyle), additional treatment may be given [8, 9].

One of the challenges for veterinarians and cat owners is that nematode infections cannot be seen. While parasites (e.g. eggs, larvae) can be detected in faeces, faecal examinations and faecal diagnostic antigen testing are not routine in general practice. However, a recent survey of cats in seven European countries showed co-infection with fleas and gastrointestinal nematodes in 11.9% of tested cats and found access to the outdoors to be a common risk factor for infection with ecto- and endoparasites [5]. While this should not replace routine faecal examination, so that parasiticide treatment is tailored to suit what the cat needs, the presence of ectoparasites can help to serve as a guide that treatment for endoparasites may also be required.

The introduction of the extended duration isoxazoline fluralaner as a topically applied spot-on product has provided cat owners with an option to provide year-round flea and tick control based on application every 12 weeks. The extended duration of activity of fluralaner provides veterinarians with a means to help improve owner compliance with parasite control recommendations, as well as substantially reducing or even removing the risk of parasite-related disease [10]. In a randomized, controlled field study in client-owned cats in the USA, there was an at least 98.6% reduction in flea counts for 12 weeks following a single topical application of fluralaner [11].

Macrocyclic lactones are a diverse family of systemically active, broad spectrum parasiticides that include both avermectins (ivermectin, selamectin and eprinomectin) and milbemycins (milbemycin oxime and moxidectin). The macrocyclic lactones have a spectrum of activity that includes insects, arachnids and nematodes, depending on the agent and species. While macrocyclic lactones have a long half-life and large apparent volume of distribution (greater than the total body water volume), meaning that they are able to penetrate tissues of the body, this varies considerably between agents [12]. Moxidectin, at a dose rate of 1 mg/kg, has been used in cats as a monthly combination product with imidacloprid (for the control of fleas) for the prevention of heartworm disease and treatment of larvae, immature and adult roundworm and hookworm, and the lungworm *Eucoleus aerophilus* (also known as *Capillaria aerophila*) [13, 14]. Now, at a minimum dose rate of 2 mg/kg, moxidectin has been combined with fluralaner into a low-volume, spot-on formulation for cats (Bravecto® Plus, MSD Animal Health). This extended duration, topical product has been approved as being safe and effective for use in cats in Europe and New Zealand, where it is indicated, depending on the geography, for the treatment of flea, tick and ear mite infestations, for the prevention of heartworm disease caused by *Dirofilaria immitis*, and for the treatment of nematode (roundworm, hookworm and lungworm) infections in cats [15, 16]. A field study in Europe demonstrated that a single application of fluralaner plus moxidectin to client-owned cats provided 12 weeks efficacy against fleas and ticks that was superior to the flea control provided by three consecutive monthly applications of fipronil, and non-inferior to fipronil in controlling ticks [17]. To assess the internal parasite control provided by this formulation for cats, a Good Clinical Practice (GCP) compliant field study was initiated in four countries in Europe, Albania, Bulgaria, Germany and Hungary, to evaluate the safety and efficacy against natural infections of gastrointestinal nematodes. In this study, the nematode control provided by a single application of fluralaner plus moxidectin was compared with that provided by a single application of emodepside plus praziquantel (Profender®, Bayer Animal Health).

## Methods

### Study design

This multi-center study was conducted from June through December, 2015. Cat owners provided informed consent prior to the enrollment of any cat into the study and the initiation of treatment. Individuals involved in treatment assignments and in treatment administration were not masked during the study and were not involved in clinical assessments. The study personnel making clinical observations and parasitologists examining faecal samples were masked to treatment assignments.

### Animals

Healthy cats, at least 10 weeks old and weighing at least 1.2 kg, were eligible for inclusion. To qualify, a positive faecal worm egg count was required, collected no more than eight days prior to the day of treatment, for gastrointestinal nematode parasites (roundworms, and/or hookworms, or other). Cats with a chronic medical condition could be included at the discretion of the Investigator. Cats could not have been treated with any anthelmintic or endectocide within 14 days before the start of the study, and treatment with any other drug from these classes was not permitted during the study. Evidence of skin disease, either generalized or at the intended product application site, was a basis for exclusion from the study, as were pregnancy or lactation. Routine health procedures, such as vaccination, and medical care were permitted. Cats were maintained by owners in the home environment or were retained in an animal shelter. If needed, cats were housed individually at the study site or in the animal shelter for collection or correct identification of faecal samples.

### Randomization and treatment

Cat enrollment numbers, set in accordance with current anthelmintic evaluation guidelines, planned for a 2:1 ratio randomization of 134 cats to the fluralaner plus moxidectin group and 67 to the emodepside plus praziquantel group [18]. With an estimated drop-out rate of approximately 10%, the number of cats to be included was therefore 150 in the fluralaner plus moxidectin group and 75 in the emodepside plus praziquantel group. In order of presentation at each site, cats eligible for inclusion were stratified by the Investigator into blocks of three and randomly allocated to a treatment group, using computer generated randomization lists. Treatment was administered on a single occasion, on Day 0, taking care to avoid product run-off by parting the hair and applying it directly to the skin, in one or two spots in an area from the base of the skull to between the shoulder blades. The minimum dose rates were 40 mg fluralaner plus 2 mg moxidectin/kg and, according to the manufacturer’ instructions, 3 mg emodepside plus 12 mg praziquantel/kg.

### Faecal egg counts

Cats were presented for faecal sampling up to 8 days prior to and 14 ± 4 days after treatment. Faecal samples from each cat were either sent to a local designated laboratory or were examined on site. Nematode eggs were counted using the modified McMaster method. For flotation, either zinc sulphate solution (Albania, Hungary), sodium chloride solution (Bulgaria), or zinc chloride/sodium chloride solution (Germany) was used. Flotation solutions were adjusted to a specific gravity of 1.3 in Germany and Albania and 1.18 in Bulgaria and Hungary. The sensitivity of counting techniques was 25 (Albania) or 50 eggs per gram of faeces (epg) (Bulgaria, Hungary, Germany). The amount of faeces used for each test was 2 g (Bulgaria), 3 g (Albania, Hungary) or 4 g (Germany). Identification of parasites was based on the distinct morphology of the ova found in the faeces. Hookworms were identified to genus and species in Albania and Germany but not in Bulgaria and Hungary.

### Efficacy assessment

The efficacy of each product was calculated for all of the cats that received at least one treatment and were examined according to the protocol (per-protocol (PP) population). The safety of each product was assessed for all cats that were treated (intent-to-treat (ITT) population). The statistical unit was the individual cat. Homogeneity of study groups at inclusion was evaluated descriptively in both the ITT and PP populations as a confirmatory indicator of the quality of randomization and allocation of cats to treatment groups. Pre-treatment means (Day 0) for individual age and weight were calculated for both study groups, as were means, standard errors, minima, and maxima for faecal egg counts (FEC) of each nematode genus and/or species. The comparison of FEC distributions by nematode species was restricted to those animals positive for the respective nematode. Frequency tables were used to compare the distribution of sex, breed and living conditions in both study groups.

The primary efficacy criterion for each group was the percent reduction in FEC for each nematode genus and/or species calculated for geometric and arithmetic means using the formula:

$$ \mathrm{Efficacy}\;\left(\%\right)=\left({\overline{\mathrm{X}}}_{\mathrm{g}\;\left(\mathrm{pre}-\mathrm{treatment}\right)}-{\overline{\mathrm{X}}}_{\mathrm{g}\left(\mathrm{post}-\mathrm{treatment}\right)}\right)/{\overline{\mathrm{X}}}_{\mathrm{g}\left(\mathrm{pre}-\mathrm{treatment}\right)}\times 100 $$

where X_g_ is the mean FEC of each group. To address possible zero counts, the geometric means were calculated as follows:

$$ {\mathrm{X}}_{\mathrm{g}}={\left(\prod \limits_{\mathrm{i}=1}^{\mathrm{n}}\left({\mathrm{X}}_{\mathrm{i}}+1\right)\right)}^{\frac{1}{\mathrm{n}}}-1 $$

To compensate for the skewed distribution, the FEC was log-transformed and shifted prior to the statistical test: x_i_' = log (x_i_ + 1). Pre- and post-treatment FECs were compared using a two-sided, two sample t-test for paired samples (*α* = 0.05). Efficacy for a nematode species was claimed if a FEC reduction of at least 90% was demonstrated for each nematode species in 10 cats that were initially positive for that species, and if there was a significant difference between pre- and post-treatment FEC.

Secondary efficacy was based upon the proportion of cats that had FECs of zero post-treatment. Non-inferiority was determined by comparing the percent of nematode-free cats after treatment with fluralaner plus moxidectin with the percent nematode free after treatment with emodepside praziquantel. The Farrington-Manning test of non-inferiority for the risk difference was used with a level of significance of α = 0.025 and a tolerated difference of δ = 0.15 [19]. Both *P*-value and lower 97.5% one-sided confidence limits were calculated. If the lower confidence limit was above -0.15, non-inferiority was concluded. If the lower confidence limit was above 0, superiority of fluralaner plus moxidectin over emodepside plus praziquantel was concluded.

## Results

Faecal samples from 838 cats were screened and 273 cats (32.6%) had positive FECs. There were 182 cats included in the fluralaner plus moxidectin group, and 91 cats in the emodepside plus praziquantel group. In the ITT population there were 50 cats from Albania, 60 from Bulgaria, 65 from Germany and 98 from Hungary. More than 90% of enrolled cats in each group were described as either domestic or European, and 61.5% were two years of age or less (11.7% were older than five years), with cats from 11 or 12 weeks of age up to 15 years enrolled in both groups. Ten cats from the ITT population were excluded from the PP population: five cats because the pre-treatment sample was negative (should not have been enrolled), two because of a protocol deviation, one was lost to follow up, and one died in a road traffic accident.

There was homogeneity between the groups for both the ITT and PP populations. In the fluralaner plus moxidectin group, 23% of cats were reported as indoor only, while the equivalent number for the emodepside plus praziquantel group was 14%. For cats reported to be outdoor only, the proportions were 37% and 41%, respectively. The remainder of cats (41% and 45%, respectively) were reported by their owners to spend time both indoors and outdoors. Mean body weights were 4.2 kg and 4.1 kg, respectively, with a minimum of 1.2 kg in each group, and maximum of 6.6 kg in the fluralaner plus moxidectin group and 5.5 kg in the emodepside plus praziquantel group. Males comprised 49% of cats in the fluralaner plus moxidectin group and 53% of cats in the emodepside plus praziquantel group, and neutered cats comprised 28% and 31% of cats in the fluralaner plus moxidectin and emodepside plus praziquantel groups, respectively.

From 273 positive pre-treatment faecal samples the most commonly identified nematode was *T. cati*, eggs of which were identified in 79.1 and 82.4% of the cats in the fluralaner plus moxidectin and emodepside plus praziquantel groups, respectively. The maximum FEC for *T. cati* was 10,100 epg in the fluralaner plus moxidectin group, and 5700 in the emodepside plus praziquantel group (Table 1). Prior to treatment 8.2% of cats in the fluralaner plus moxidectin group and 6.6% of the emodepside plus praziquantel group tested positive for *Toxascaris leonina*. Hookworms were found in 30.8 and 24.2% of cats, respectively. The clinics in Germany and Albania, where hookworms were differentiated to genus and species level, showed that *Ancylostoma tubaeforme* was the most commonly identified genus, found in 21 cats in the fluralaner plus moxidectin group and five cats in the emodepside plus praziquantel group. *Uncinaria stenocephala* was found less commonly; in five and three cats, respectively. *Capillaria* spp. were found in 7.1 and 4.3% of cats, respectively.

**Table 1:** Geometric (arithmetic) mean faecal egg counts and percent reduction (per protocol population)

Group	Species	Pre-treatment		Post-treatment		Efficacy (%)
		Range	Mean	Range	Mean	
Fluralaner / moxidectin	*Toxocara cati*	25–10,100	407.29 (788.38)	0–1100	0.13 (9.86)	99.97 (98.75)
	*Toxascaris leonina*	50–2000	126.36 (250.00)	0	0 (0)	100 (100)
	*Capillaria* spp.	50–800	126.09 (173.21)	0	0 (0)	100 (100)
	Hookworm spp.^a^	50–11,400	252.01 (660.91)	0	0 (0)	100 (100)
Emodepside / praziquantel	*Toxocara cati*	50–5700	280.90 (591.78)	0–11,950	0.20 (164.38)	99.93 (72.22)
	*Toxascaris leonina*	75– 250	155.47 (170.83)	0	0 (0)	100 (100)
	*Capillaria* spp.	50–150	95.96 (106.25)	0	0 (0)	100 (100)
	Hookworm spp.^a^	50–1650	201.12 (329.55)	0	0 (0)	100 (100)

^a^Hookworm spp. include *Ancylostoma tubaeforme* and *Uncinaria stenocephala*

Treatment was followed by a reduction in the geometric mean FEC for *T. cati* of 99.97% in the fluralaner plus moxidectin and 99.93% in the emodepside plus praziquantel groups (Table 1). In fact, *T. cati* were the only nematode eggs found in faeces from both groups after treatment. For arithmetic means, the respective reductions in *T. cati* ova were 98.75 and 72.22%, with the substantially lower efficacy in the emodepside plus praziquantel group due to the count in one cat with an FEC of 11,950 epg. Both products were 100% effective in eliminating hookworm ova. In the fluralaner plus moxidectin group, the FECs two weeks after treatment were significantly lower for each parasite (*P* < 0.0001) than baseline counts (Table 2). The primary objectives of (i) at least 90% efficacy per nematode species in 10 cats that were positive for a nematode species before treatment, and (ii) significant differences from baseline for all nematode species were met by fluralaner plus moxidectin treatment. In the fluralaner plus moxidectin group, 98.3% of cats were free of ova of all nematode species that had been present prior to treatment (Table 3). The percent of nematode-free cats in the fluralaner plus moxidectin group was significantly non-inferior to that in the emodepside plus praziquantel group (96.5%). In addition, the lower 97.5% one-sided confidence limit was well above the non-inferiority limit of -0.15, thereby meeting the secondary efficacy objective of the study (Table 3).

**Table 2:** Number of cats with positive faecal egg counts and, for the fluralaner-moxidectin group, comparison of pre- and post-treatment faecal egg counts, per protocol population

Group	Nematode species	Number positive		Difference post- *vs* pre-treatment^b^		Probability
		Pre-treatment	Post-treatment	Mean	SE	
Fluralaner / moxidectin	*Toxocara cati*	142	3 (97.89%)	5.8920	0.1130	*t*_(141)_ = 52.13; *P* < 0.0001
	*Toxascaris leonina*	15	0 (100%)	4.8470	0.2520	*t*_(14)_ = 19.24; *P* < 0.0001
	*Capillaria* spp.	14	0 (100%)	4.8449	0.1996	*t*_(13)_ = 24.28; *P* < 0.0001
	Hookworm spp.^a^	55	0 (100%)	5.5334	0.1534	*t*_(54)_ = 36.07; *P* < 0.0001
Emodepside / praziquantel	*Toxocara cati*	73	2 (97.26%)			
	*Toxascaris leonina*	6	0 (100%)			
	*Capillaria* spp.	4	0 (100%)			
	Hookworm spp.^a^	22	0 (100%)			

^a^Hookworm spp. include *Anyclostoma tubaeforme* and *Uncinaria stenocephala*

^b^Differences not calculated for emodepside/praziquantel

*Abbreviation*: SE, standard error

**Table 3:** Percent of nematode free cats, with non-inferiority analysis at the post-treatment assessment (per protocol population)

	Presence of faecal eggs		97.5% lower confidence limit	*Z-*value	*P*-value
	Yes	No			
Fluralaner / moxidectin	3 (1.70%)	173 (98.30%)	-0.0964	3.2899	0.0005
Emodepside / praziquantel	3 (3.45%)	84 (96.55%)			

Margin = 0.15; Farrington-Manning method

The presence of eggs of the lungworm species *Capillaria* spp. in the faeces of study cats allowed assessment of efficacy against this parasite. The results indicate that both fluralaner plus moxidectin and emodepside plus praziquantel were 100% effective in eliminating *Capillaria* spp. ova from the faeces of infected cats.

Both products were well tolerated by cats. Six mild adverse events were reported during the study, four in the fluralaner plus moxidectin group and two in the emodepside plus praziquantel group. Two of the four events (mild alopecia at the application site in one cat and a small area of whitish discoloration of the hair on the neck in one cat) in the fluralaner plus moxidectin group (1% of treated cats in this group) were considered to be probably related to treatment. The other two events involved the withdrawal of the cat that died in a road traffic accident and one cat that developed diarrhea. The two cats from the emodepside plus praziquantel group had mild diarrhea that was considered to be unrelated to treatment. No serious, treatment-related adverse events occurred in either treatment group.

## Discussion

The results of the present study in Europe demonstrate that a single treatment with fluralaner plus moxidectin is highly effective against nematodes that infect cats. The findings are consistent with earlier reports that roundworm and hookworm infections are common in cats (approximately 3 of every 10 cats screened had nematode eggs in faeces), with the roundworm *T. cati* being the most prevalent [1–7]. The study showed that the efficacy of fluralaner and moxidectin was non-inferior to emodepside plus praziquantel. The results of this field study are in line with and further confirm those of a laboratory study in which a topical formulation of imidacloprid in combination with moxidectin (1%) was shown to be effective against experimental infections with *T. cati* and larval stages of *A. tubaeforme* [13].

Infection with respiratory nematodes occurs in cats in Europe. *Aelurostrongylus abstrusus* (3–5% of surveyed cats) and *Capillaria* spp, (1–2%), in particular *Capillaria aerophila* (also known as *Eucoleus aerophilus),* have been reported to be a cause of respiratory disease, as has *Troglostrongylus brevior* [5, 6]. However, prevalence rates vary and can range from around 5 to 20%, with access to the outdoors, but not age, being a risk factor [5]. The methodology in our study focused on nematode ova and not on detecting other faecal parasite stages, and so would not have detected larvae of *A. abstrusus*. Additionally, *Capillaria* eggs found in the faeces may be produced by *C. aerophila*, parasitic in the trachea and bronchi, and/or by *C. putorii* (the cat stomach worm) which inhabits the gastrointestinal tract. Nonetheless, the finding of ova of *Capillaria* spp., not identified to species level, in faecal samples from 6.8% of cats enrolled in our study are in line with earlier reports. In the fluralaner plus moxidectin group, the faeces of 14 cats were positive for *Capillaria* spp., with a geometric mean count of 135.34 epg. The complete post-treatment elimination of these ova is consistent with earlier work showing that a topical formulation of moxidectin (combined with imidacloprid) at the dose rate of 1 mg/kg eliminated *Capillaria* spp. ova from infected cats [20, 21]. The results therefore confirm that that moxidectin is highly active against *Capillaria* spp. Similarly, in the emodepside plus praziquantel group, there were no *Capillaria* spp. ova in post-treatment faecal samples, although only four cats were positive pre-treatment when FECs (up to 150 epg) were much lower than in the fluralaner plus moxidectin group (up to 800 epg).

In our study, we did not investigate infections with species of the Dipylidiidae and Taeniidae, which may occur in up to 7% of cats in Europe [5]. The two most commonly recognized of these cestodes, *Dipylidium caninum* and less so *Taenia taeniaformis*, are typically associated with infection in the cat, which is regarded as being epidemiologically insignificant in the transmission of a third tapeworm, *Echinococcus multilocularis* [5, 8]. As neither fluralaner nor moxidectin, nor the families of isoxazolines and macrocyclic lactones to which they belong, have demonstrated evidence of activity against cestode infections, cat owners seeking control of these parasites would need to explore additional treatment options. Elimination of infections with *D. caninum* can be achieved by treatment with praziquantel, while effective flea control presents an ideal means of preventing further infection which occurs through oral ingestion of infected fleas. Praziquantel is also effective in the treatment of infection with *T. taeniaformis,* which is most likely to occur in cats that hunt on a regular basis and so are exposed through consumption of infected intermediate hosts (e.g. rodents) [8]. When treatment of feline cestode infections is required, a praziquantel-containing product administered concomitantly with fluralaner plus moxidectin has been shown to be safe [22, 23].

Regular treatment of gastrointestinal nematodes is recommended not only because of the potential clinical impact of roundworms and hookworms but also as a hygienic measure and to decrease the risk of zoonotic transmission of *T. cati* and *Ancylostoma* spp. [8]. An important benefit of the fluralaner plus moxidectin formulation for cats is that treatment at 12-week intervals not only simplifies the provision of flea and tick control but also, with approximately four treatments per annum, provides broad spectrum treatment of nematodes that is in line with the recommendations of opinion-leading organizations in this field, such as ESCCAP. In higher-risk situations, additional anthelmintic treatments can be built-in to a tailored parasite control program for an individual cat, as required. This simplifies the treatment regimen for the cat owner as well as avoiding potential over-treatment of nematode parasites that may occur when products that require monthly application for tick and/or flea control, such as the combinations of fipronil, (*S*)-methoprene, eprinomectin and praziquantel, imidacloprid and moxidectin, and selamectin with or without sarolaner, are used.

The safety of both fluralaner and moxidectin in cats, as single entity or combination products, has been well established in field and laboratory studies over years of use [11, 13, 14, 17, 20–23]. This study confirmed that fluralaner plus moxidectin (at dose rates from 40–93 mg fluralaner and 2–4.65 mg moxidectin/kg) and emodepside plus praziquantel were safe and well tolerated by cats under field conditions.

## Conclusions

This extended duration fluralaner plus moxidectin product, with 12-week efficacy against fleas and ticks, has been confirmed under field conditions to be safe and effective in the treatment of broad range of nematodes in cats.

## Abbreviations

epg: eggs per gram of faeces
ESCCAP: European Scientific Counsel Companion Animal Parasites
FEC: faecal egg count
ITT: intent-to-treat
PP: per protocol
X̅_g_: denotes the mean faecal egg count
